# A Question of Clothing

**Published:** 1908-09-26

**Authors:** 


### A Question of Clothing.

There must be very few whose memory goes back a decade or so who have not smiled at the coloured cartoons, then very popular, that appeared over the pseudonym of "Cynicus." There was moral teaching, as well as humour in many of his productions, one of the best known of which was a picture of a stout and very *décolletée* lady stepping out of a splendid carriage, whilst two tattered street arabs looked on in admiration, or, at least, in wonder. The legend underneath ran something after this fashion: "Both rich and poor alike their nakedness display, The poor because they must, the rich because they may." This genial piece of satire springs irresistibly into recollection on perusing the animated contribution which has been published in the "Journal of the Royal Institute of Public Health" as part of a controversy on the question of the hygiene of modern female apparel. Dr. Bulstrode having attacked the wearing of corsets as bad for the lungs and other organs, and having attributed the decrease in pulmonary tuberculosis to a decline in popularity of corsets, Dr. Lilian Chesney replies that women are not giving up stays, and, moreover, that the latter are not in any way injurious to health. According to Dr. Chesney, it is the suspension of garments from the shoulders which is the great hygienic evil, sartorially speaking, and is responsible for the preferential invasion of the pulmonary apices by the tubercle bacillus. She goes on to defend the custom of wearing low-necked evening dress, thin shoes and stockings, and scanty underwear at dances and other forms of evening amusement. It might well be supposed that to alternate violent exercise with exposure to draughts while only partially clad, in spells of five minutes each for half the night, is to invite pulmonary diseases; but Dr. Chesney says that, in point of fact, women very rarely catch cold in the evening: there is far less harm, she thinks, in uncovering the chest, head, etc., for a portion of the day than in coddling them the whole clock round, and she is especially insistent on the detriment of heavy woollen undergarments. The night sweats of consumptives are held to be partly induced—at least amongst the lower orders—by the unwholesome habit of wearing the day underwear during the night, and several additional garments as well.

There is probably a good deal of truth in this portion of Dr. Lillian Chesney's thesis, though we cannot say we are quite convinced that corsets are so entirely innocent of harm as she believes, or that the apical distribution of tuberculous lesions is much affected by the precise manner of wearing the clothes. As far as that goes, savages whose attire consists of a loin-cloth or a string of beads, for instance, should, on this hypothesis, be partially immune from tuberculosis, or at least not subject to the usual apical preference. But this is not, as it happens, the case in actual experience. At the same time, if more fresh air, particularly for young children; less pampering and overdressing; greater hardihood, and capacity to endure with equanimity and without ill result the vagaries of the English climate—if all these worthy ends are promoted in any degree by the advocacy of Dr. Chesney (or anyone else), we shall congratulate her, ourselves, and the British public on a result which is much to be desired. The fetish of woollen clothing and too much of it obsessed everyone towards the end of the nineteenth century; but there are encouraging signs of a reaction lately, and we are glad to see them.

